# Glucocorticoid and Estrogen Receptors Are Reduced in Mitochondria of Lung Epithelial Cells in Asthma

**DOI:** 10.1371/journal.pone.0039183

**Published:** 2012-06-27

**Authors:** Davina C. M. Simoes, Anna-Maria G. Psarra, Thais Mauad, Ioanna Pantou, Charis Roussos, Constantine E. Sekeris, Christina Gratziou

**Affiliations:** 1 “G.P. Livanos and M. Simou” Laboratories, Evangelismos Hospital, Department of Critical Care and Pulmonary Services, University of Athens Medical School, Athens, Greece; 2 University of Thessaly, Department of Biochemistry and Biotechnology, Larissa, Greece; 3 Biomedical Research Foundation of the Academy of Athens, Center for Basic Research, Laboratory of Biochemistry, Athens, Greece; 4 Department of Pathology, Sao Paulo University Medical School, Sao Paulo, Brazil; 5 National Hellenic Research Foundation, Institute of Biological Research and Biotechnology, Laboratory of Molecular Endocrinology, Athens, Greece; University of Barcelona, Spain

## Abstract

Mitochondrial glucocorticoid (mtGR) and estrogen (mtER) receptors participate in the coordination of the cell’s energy requirement and in the mitochondrial oxidative phosphorylation enzyme (OXPHOS) biosynthesis, affecting reactive oxygen species (ROS) generation and induction of apoptosis. Although activation of mtGR and mtER is known to trigger anti-inflammatory signals, little information exists on the presence of these receptors in lung tissue and their role in respiratory physiology and disease. Using a mouse model of allergic airway inflammation disease and applying confocal microscopy, subcellular fractionation, and Western blot analysis we showed mitochondrial localization of GRα and ERβ in lung tissue. Allergic airway inflammation caused reduction in mtGRα, mtERβ, and OXPHOS enzyme biosynthesis in lung cells mitochondria and particularly in bronchial epithelial cells mitochondria, which was accompanied by decrease in lung mitochondrial mass and induction of apoptosis. Confirmation and validation of the reduction of the mitochondrial receptors in lung epithelial cells in human asthma was achieved by analyzing autopsies from fatal asthma cases. The presence of the mitochondrial GRα and ERβ in lung tissue cells and especially their reduction in bronchial epithelial cells during allergic airway inflammation suggests a crucial role of these receptors in the regulation of mitochondrial function in asthma, implicating their involvement in the pathophysiology of the disease.

## Introduction

Asthma is an inflammatory lung disease with airway hyperresponsiveness (AHR) [1]. The inflammatory cells release reactive oxygen species (ROS), which leak into surrounding cells [2], [3]. The produced oxidative stress causes respiratory epithelial cell damage, affecting the first line of defence against inhaled agents including allergens [2], [3], [4]. In fact, bronchial epithelial cells from asthmatics have greater susceptibility to oxidants [5]. However, in spite of many epidemiological studies [6]–[8] and investigation on the mechanisms mediating the effect of oxidants on the pathophysiology of asthma, their exact role in the disease process is unknown [9].

Glucocorticoids (CS), the mainstay treatment of asthma, act by activating glucocorticoid receptors (GR). Steroid receptors are a major class of nuclear receptors representing ligand-activated transcription factors, known to regulate many cellular functions, including inflammatory process, energy production and apoptosis [10]. In these processes mitochondria play a major role integrating a variety of intracellular and extracellular signals including apoptotic signals [11]–[13]. Thus, although CS therapy suppresses allergen-induced airway inflammation, epithelial cell shedding is not corrected [14], [15]. In fact, CS treatment induces mitochondrial depolarization and activation of caspase-9 leading to airway epithelium apoptosis [14]. In addition, CS administration down-regulates GRα in bronchial epithelial cells [16], [17], and pro-inflammatory stimuli decrease GR binding activity and steroid responsiveness. However, the mechanism of CS elicitation of epithelial cell damage and apoptosis is not understood.

Mitochondria provide 90% of the cell energy via oxidative phosphorylation, catalysed by five membrane-bound protein complexes (Complex I to V), and generating the majority of the cell’s ROS. The subunits components of these OXPHOS complexes are encoded by both mitochondrial and nuclear genes with the exception of complex II, only encoded by nuclear genes [18]. These complexes are synthesized by tightly coordinated and integrated process [13], [18]. Thyroid, glucocorticoid and estrogen receptors (ER) participate in this tight coordination acting as transcription factors for both nuclear and mitochondrial-encoded OXPHOS gene expression [13], [19]–[21]. Mitochondrial GR and ER have been found in mitochondria of certain cell types (reviewed in [13], [21]). Putative hormone response elements (HRE) for GR and ER have been detected in the mitochondrial genome [22]. The ability of GR to bind to HRE located on the regulatory D-loop region of the mitochondrial genome of hepatoma cells [23], [24] and to directly regulate mitochondrial transcription and OXPHOS enzyme biosynthesis has been demonstrated [24], [25]. Binding of recombinant ERβ to mitochondrial DNA of MCF-7 cells has also been documented by EMSA analysis [26].

**Figure 1:**
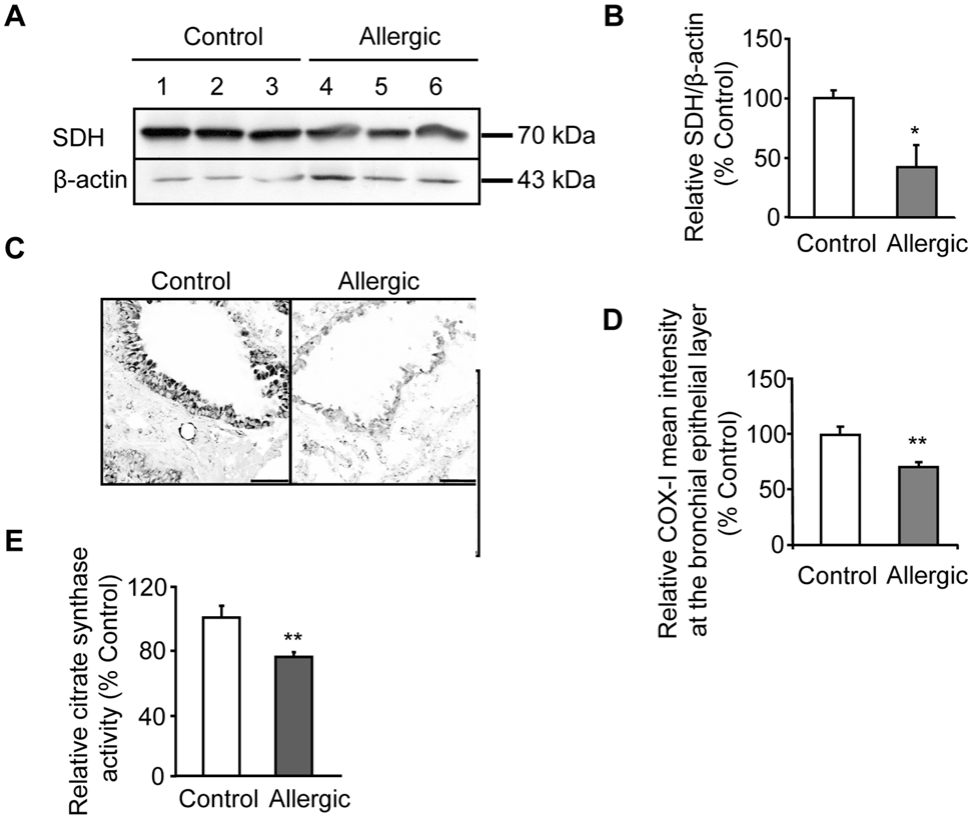
Lung mitochondria are reduced by allergic airway inflammation. Mitochondria was assessed in lung of mice sensitized with Ova and alum (allergic) compared to healthy controls (PBS/alum sensitization) and subsequently challenged with Ova as described in Methods section. (A) Expression of the mitochondrial succinate-ubiquinol oxidoreductase subunit (SDH) component of the OXPHOS Complex II was studied by Western blot in total fresh lung homogenates. Representative Western blots are shown. (B) Quantitation of band intensity by densitometry from blots after normalization against actin is shown. (C) Representative images of immunostained frozen lung sections from control and allergic mice for mitochondrial cytochrome c oxidase subunit-I (COX-I) component of OXPHOS Complex IV depicting mitochondria cell distribution, as described in Methods section. (D) Quantitation of relative intensity of COX-I in bronchial epithelial selected using lasso tool were analyzed using Leica LAS-AF image analysis system as described in Methods. (E) Citrate synthase activity was measured in total homogenates from allergic mice (n = 5) and expressed as means ± S.D. of percentage of activity in total homogenates from control mice (n = 3). Activity was set at 100% for controls. Results are presented as means ± SEM; relative expression of SDH and COX-I mean intensity was set at 100% for controls. n = 10–12 mice per group; *p<0.05 from control, **p<0.001 from control. Scale bars, 50 µm.

However, the presence of mtGR and mtER in lung tissues has not been studied. Although derangements of mitochondrial function in asthma have been observed [27]–[29], even in the context of CS treatment, a possible association has not be demonstrated.

All the above led us to evaluate the presence of GR and ER in mitochondria of lung epithelial cells in rats and human tissues and to explore their possible role in allergic airway inflammation. In this study, we present evidence showing localization of GR and ERβ in lung epithelial cells. Allergic airway inflammation caused decrease in mitochondrial steroid receptors expression, which was accompanied by decrease in lung mitochondrial mass, OXPHOS enzyme biosynthesis and induction of apoptosis. We propose that the decreased expression of steroid receptors in mitochondria of bronchial epithelial cells by allergic airway could affect mitochondrial function and trigger a series of event crucial for the final outcome of the disease.

**Figure 2:**
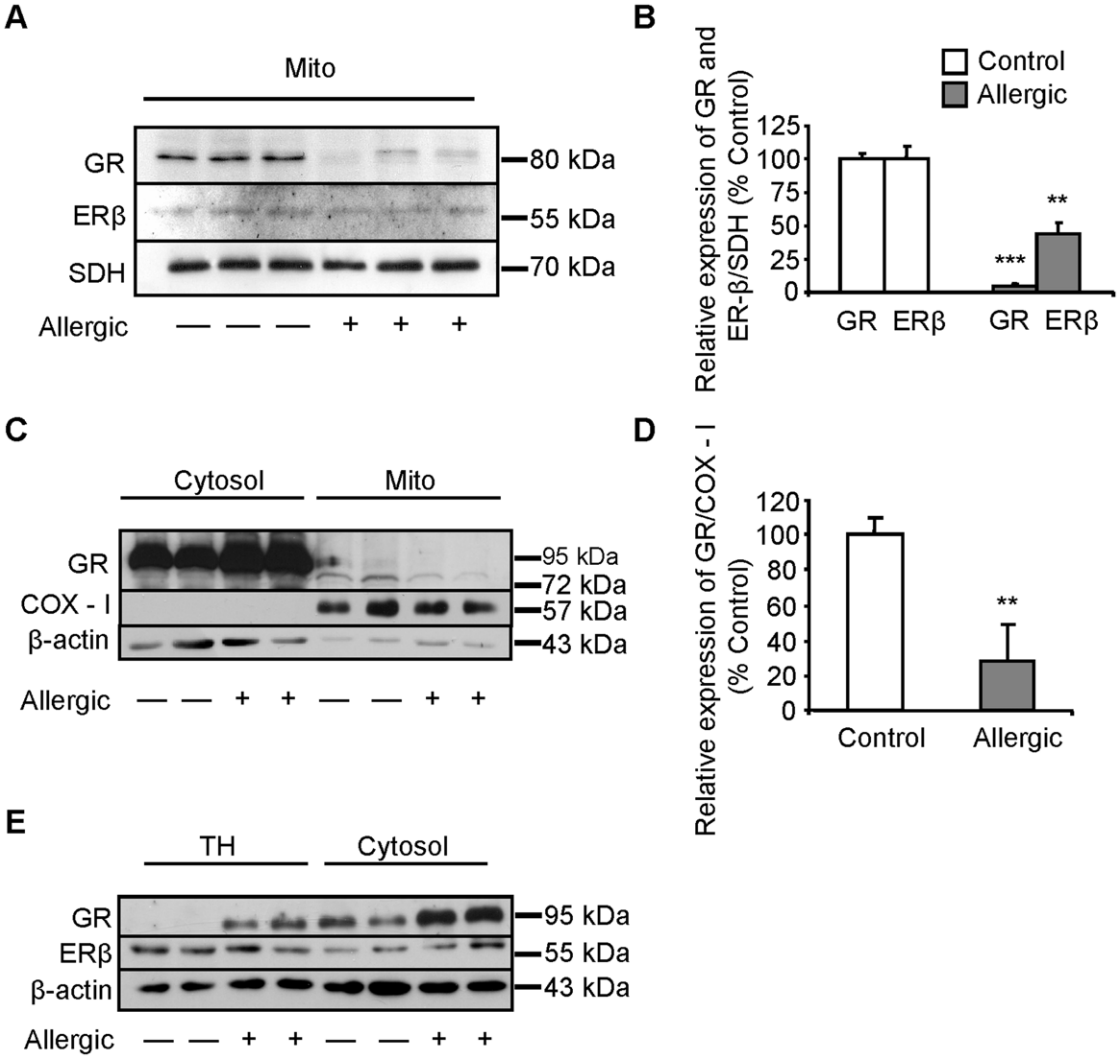
Allergic airway inflammation reduces GR and ERβ in highly purified mitochondrial fraction. Highly purified mitochondria were isolated from total lung homogenates from allergic and control mice as described in Methods section. (A) Representative Western blot images showing mtGR and mtERβ in isolated mitochondrial fraction. (B) Quantitation of band intensity by densitometry from blots after normalization against the succinate-ubiquinol oxidoreductase subunit of the mitochondrial Complex II OXPHOS enzyme (SDH). (C) Western blot analysis of GR, COX-I, and β-actin protein levels in cytosolic (Cytosol) and mitochondrial (Mito) fractions from control and allergic mice. (D) Quantitation of mtGR band intensity by densitometry after normalization against the expression levels of COX-I protein. (E) Western blot analysis of GR, ERβ, and β-actin, in total homogenates (TH) and cytosolic fractions from control and allergic mice. Results are presented as means ± SEM; relative measurements of GR and ERβ were set at 100% for control mice. n = 10–12 mice per group; **p<0.001; ***p<0.0001 from control.

## Materials and Methods

### Mice Allergic Airway Inflammation Assessment

BALB/c mice were sensitized intraperitoneally with ovalbumin (Ova) in alum on days 0 and 12 and assessed as previously described [30], [31] (Details about experimental protocols see: Supporting Information S1). Allergic airway inflammation was induced by aerosolized (5%) Ova (days 18–23). Mice were housed at the Experimental Surgery-Unit of Evangelismos Hospital. All procedures were approved by the Veterinary Administration Bureau of the Prefecture of Athens, Greece, and were in accordance with the US National Institutes of Health Statement of Compliance (Assurance) with Standards for Humane Care and Use of Laboratory Animals (#K-2654), and with the European Union Directive 86/609/EEC for animal research.

**Figure 3:**
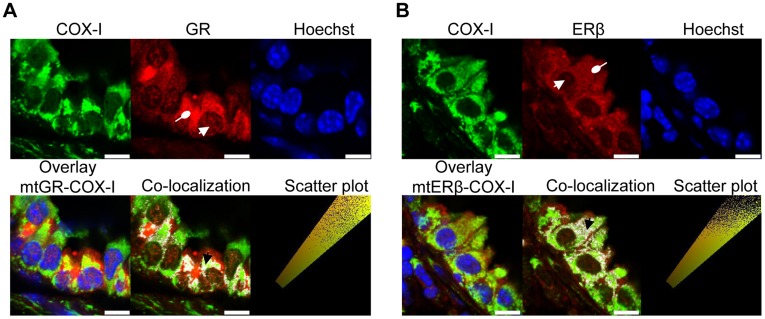
Cells from bronchial epithelial layer express both GR and ERβ receptor in mitochondria. Representative immune-fluorescent images of antibodies against COX-I (green), GR (red), ERβ (red) and Hoeschst (nuclear fluorescence stain, blue) were obtained with confocal laser scanning microscopy. Yellow co-localized pixels in overlay images for (A) mtGR-COX-I and, (B) mtERβ-COX-I are depicted. Co-localisation analysis and quantification were performed using the Leica Las-AF image system, where background and threshold were set, generating a colocalization image (colocalized pixels in white; black arrow head) and a pixels distribution scatter plot (co-localized yellow pixels along the diagonal). White point arrow head shows nuclear distribution and white rounded arrow head shows cytoplasmic distribution. Scale bars, 7.5 µm.

### Human Subjects

Autopsies from five fatal asthma cases (3 females and 2 males, with a median age of 45 years and an age range of 33–55 years), with previously known history of asthma, and from five control cases (4 females and 1 male, with a median age of 55 years and an age range of 46–63 years), were obtained from formerly studied population [32]. Human lung tissues were surgically removed within the last four years, immediately fixed in 10% neutral phosphate-buffered formalin for 24–48 hr, routinely processed, and paraffin embedded. Mean time between death and autopsy was 12,4 hours (range: 9–19 hours) for controls and 13,2 hours (range: 11–18 hours) for asthmatics. Controls were non-smokers subjects, with no previous pulmonary diseases, that died of non-pulmonary causes, mostly due to acute cardiovascular diseases, and had normal lungs after histological examination. Written consent from relatives to use the autopsy material and the clinical information were obtained via questionnaires in research studies. Approval for the use of these samples in research was obtained from the review board for human studies of the School of Medicine, Sao Paulo University.

### Subcellular Fractionation for the Isolation of Highly-purified Mitochondria

Fresh lungs (3 mice) were pooled together, homogenized, and subjected to isolation of cytosol and mitochondrial fraction using discontinuous sucrose-gradient as previously described [33] (Details about experimental protocols see: Supporting Information S2). Samples were kept at −80°C.

**Figure 4:**
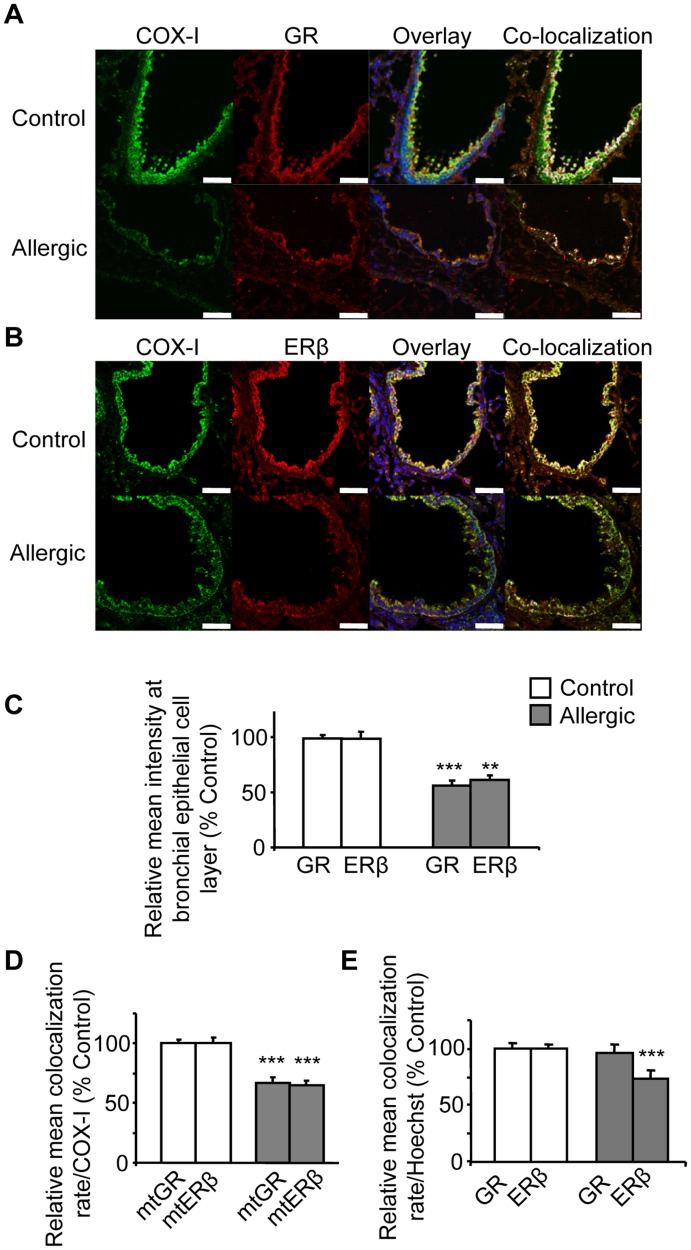
Allergic airway inflammation reduces mtGR, mtER and nuclear ER in mice lung epithelial cell layer. Confocal immunofluorescence images of control and allergic mice lung sections immunostained for COX-I (green), GR (red), ERβ (red) and Hoeschst (nuclear fluorescence stain, blue) were obtained and analysed using Leica Las-AF image system. (A) Representative images of COX-I staining, GR staining, overlay mtGR-COX-I-Hoeschst, and analysed co-localized mtGR-COX-I white pixels are depicted. (B) Representative images of COX-I staining, ERβ staining, overlay mtERβ-COX-I-Hoeschst, and analysed co-localized mtERβ-COX-I white pixels are depicted. (C) Mean intensity of immunostaining for GR and ERβ, in regions-of-interest (ROIs) placed at epithelial cell layer, were quantified using Leica Las-AF image analysis system as described in Methods section. Mean percentages of co-localization rate with COX-I (D) and Hoechst (E) were calculated at ROIs placed at epithelial cell layer in allergic mice relative to controls as described in Methods section. Results are presented as means ± SEM; GR and ERβ percentage of co-localization rate was set at 100% for control mice. n = 10–12 mice per group; ***p<0.0001 from control. Scale bars, 50 µm.

### Western Blotting

Expression of the nuclear encoded mitochondrial succinate-ubiquinol oxidoreductase subunit (SDH) of the OXPHOS Complex II, the mitochondrial encoded cytochrome oxidase subunit I (COX-I) of Complex IV, GR, ERβ, and β-actin, was studied in cellular and subcellular fractions of lung tissues from control and allergic mice applying Western blot analysis. In addition, mitochondrial apoptotic signalling pathway was accessed by analysing the protein levels of caspase-9 and cytochrome-c in the cytosol, and of poly ADP-ribose polymerase (PARP) and cleaved caspase-3 in total lung homogenates. Immunoblotting conditions and antibodies utilized are as follow anti-GR (M-20) (1∶500; Santa Cruz Biotechnology); anti-ERα (MC-20; Santa Cruz Biotechnology) (1∶250); anti-ERβ (H-150; Santa Cruz Biotechnology) (1∶500); mouse anti-succinate-ubiquinol oxidoreductase 70 kDa subunit of complex II (SDH) (1∶5000; Invitrogen); mouse anti-COX-I (2 µg/ml; Invitrogen) and mouse anti-actin (1∶10000; Sigma-Aldrich). For apoptosis induction assesment anti-cytochrome C (7H8) (1∶500; Santa Cruz Biotechnology), anti-caspase-9 (1∶10000; Cell Signalling), anti-caspase-3 (cleaved) (1∶500, Cell Signalling), anti anti-PARP (1∶1000; Sigma) antibodies were used. Specificity of steroid receptors antibodies has been proved by the use of commercially provided specific blocking peptides [33], [34].

**Figure 5:**
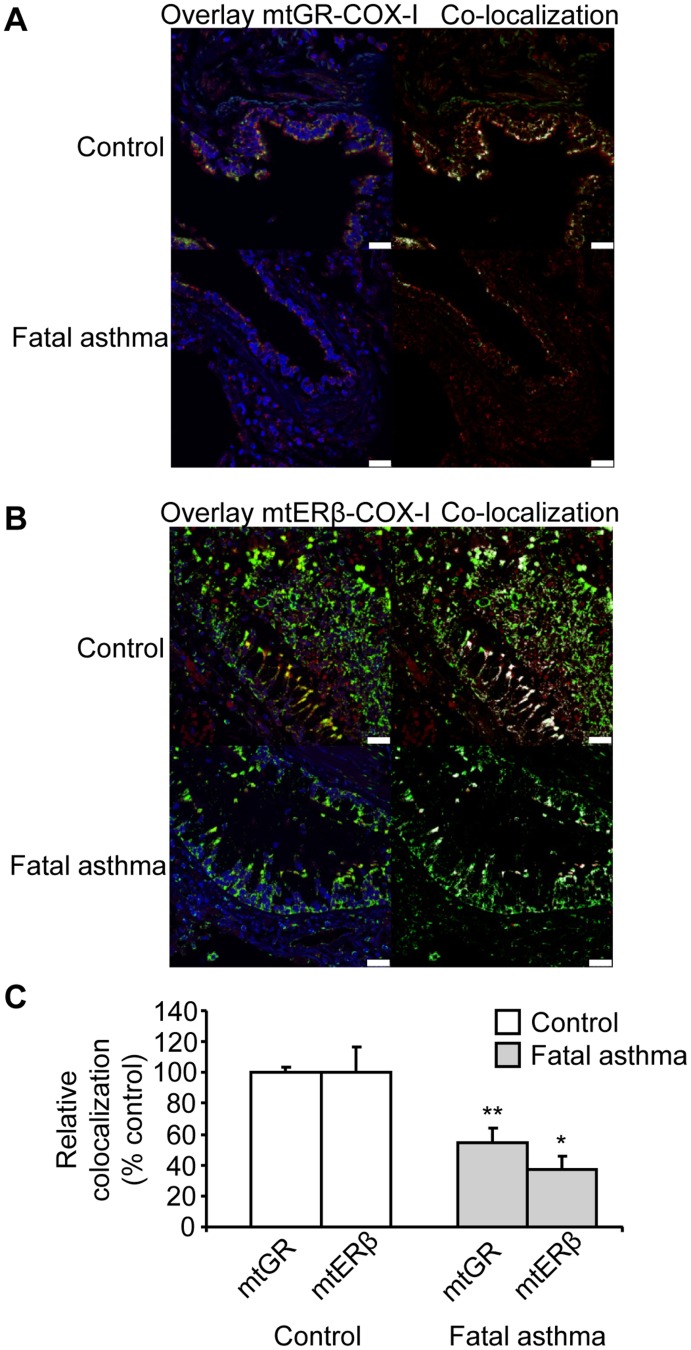
mtGR and mtERβ are reduced in autopsies from fatal asthma patients. Confocal laser scanning microscopy images from control cases and fatal asthma patients were analysed using Leica Las-AF image system. Representative overlay and co-localized pixels (white pixels) images of human lung sections immune-stained with antibodies against (A) COX-I (green) and GR (red) and (B) COX-I (green) and ERβ (red) are depicted. Sections were also stained with Hoeschst nuclear fluorescence stain (blue). (C) Mean percentages of co-localization rate were calculated at ROIs placed at epithelial cell layer in the human autopsies as described in Methods section. Results are presented as means ± SEM; mtGR and mtERβ percentage of co-localization rate set at 100% for control subjects. n = 12–20 bronchi from autopsies per group; *p<0.05 from control; **p<0.001 from control. Scale bars, 25 µm.

### Immunofluorescence and Confocal Laser Scanning Microscopy Analysis

Human and mice lung sections (4 µm) were fixed by applying methods providing preservation of mitochondrial structures, assessed by COX-I staining. Briefly, re-hydrated paraffin or frozen lung sections sections (4 µm) were placed in 50 mM Tris-HCl buffer (pH 7.5) at 95°C for 20 min and fixed in 2% paraformaldehyde (PFA) in phosphate buffer saline (PBS) at room temperature (RT) for 20 min. After three washings (5 min each) with PBS, sections were blocked with 10% donkey/horse serum with 0.1% Triton for 20 min at RT. Subsequently, sections were immunostained with antibodies against COX-I conjugated to Alexa-Fluor-488 (1∶200; Molecular Probes); and GR-M20 or ERβ-H-150 (1∶50; Santa Cruz Biotechnology) or cleaved-caspase-3 (1∶200; Cell Signaling) complexed with secondary goat-anti-rabbit antibodies conjugated to Alexa-Fluor-568 (1∶500). Following three washes, sections were incubated with 1 µg/ml Hoechst in PBST, for 5 min at room temperature. After being rinsed several times in PBS, the specimens were mounted in antifading medium. Triple-stained images were obtained with confocal microscopy (Leica TCS SP5) and analysed as described in Supporting Information S3.

**Figure 6:**
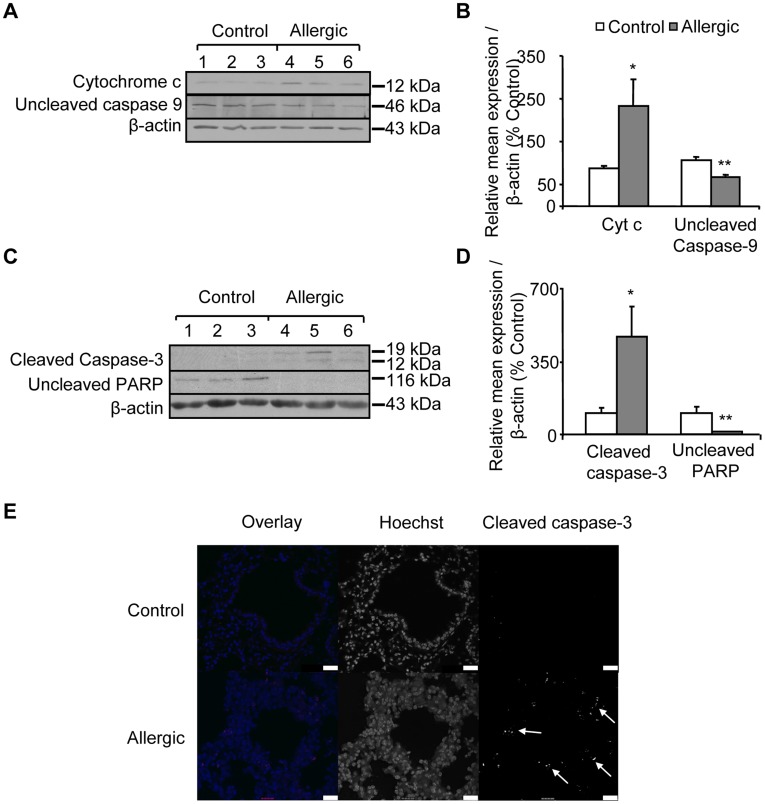
Allergic airway inflammation increases pro-apoptotic signalling from mitochondria. Cytosol from control and allergic mice were isolated during the subcellular fractionation procedure as described in Methods section. (A) Expression of the uncleaved caspase-9 and cytochrome c were studied by Western blot in the cytosol. Representative Western blots are shown. (B) Quantitation of band intensity by densitometry from blots after normalization against actin is shown. (C) Expression of the PARP and cleaved caspase-3 were studied by Western blot in total lung homogenate. Representative Western blots are shown. (D) Quantitation of band intensity by densitometry from blots after normalization against actin is shown. (E) Hoechst and anti-cleaved caspase-3 (red) staining in representative confocal images from control and allergic mice lung sections. White arrow heads points to cleaved caspase-3 shown as red pixels in overlaid cleaved caspased-3-Hoechst images and as white pixels in cleaved caspase-3 grey scale images. Scale bars, 25 µm. Results are presented as means ± SEM; *p<0.05; **p<0.001 from control.

### Citrate Synthase Activity

Citate synthase activity in total homogenates from control and allergic mice was spectrophotometrically measured at 412 nm, in reaction buffer containing: Tris – HCl pH: 8.0; 0.1 mM acetyl-CoA, 0.2 mM 5,5′-Dithio-Bis (2-Nitrobenzoic Acid) (DTNB), 0.05 mM oxalic acid, as previously described [35].

### Statistical Analysis

Results are presented as means ± SEM. Comparisons were made either by t-test or by analysis of variance followed by Tukeys’s post-hoc test using SPSS software. Differences were considered significant when p<0.05.

## Results

### Lung Mitochondria are Reduced during Allergic Airway Inflammation

Mice sensitized and challenged with Ova (allergic), as described in methods section, displayed many of the characteristics of asthma (Figure S1). BAL fluid (Figure S1A) and histological sections stained with Hematoxylin and Eosin (H&E) (Figure S1B) confirmed large number of inflammatory cells, which was accompanied by increased mucus production (Figure S1B). In addition, lung homogenates from allergic mice had significantly increased expression of IL-4 by 11.4 pg/ml compared to non-allergic controls (Figure S1C). The above characteristics were accompanied by increased airway Newtonian resistance, an index of bronchoconstriction and airway hyperresponsiveness (Figure S1D).

The nuclear encoded mitochondrial SDH component of the OXPHOS Complex II, when normalised against actin, was significantly reduced in total lung homogenates of allergic mice compared to controls (Figure 1A and 1B). Likewise, the mitochondrial encoded COX-I component of OXPHOS Complex IV was also significantly reduced (by approximately 27%) at the bronchial epithelial cell layer of allergic mice compared to controls (Figure 1C, 1D), as indicated by the mean immunofluorescence intensity obtained. Accordingly, citrate synthase activity in total homogenates from allergic treated mice was reduced compared to controls (Fig. 1E), indicating that allergic airway inflammation reduced the amount of mitochondria in total lung homogenate.

### Allergic Airway Inflammation Reduces mtGR and mtER at the Bronchial Epithelial Cell Layer

GR and ERβ expression levels were examined in cellular and subcellular fractions (total homogenate, cytosolic, and mitochondrial fraction) of lung tissues from control and allergic mice.

Equal protein amounts of highly purified mitochondria isolated from total lung tissue showed the presence of an 80 kDa GR band recovered from allergic mice, which represented a significantly reduced amount of a same size GR band observed in control mice (Figure 2A and 2C). Mitochondrial SDH as well as COX-I expression levels (Figure 2B and 2D) were used for the normalization of the results. ERβ was also found in the mitochondrial fractions from control and allergic mice exhibiting reduction in allergic mice, which was less striking compared to mtGR reduction (Figure 2A and 2B). The ERα isoform was not detected in the mitochondrial fraction (data not shown). GR was increased in total homogenate and cytosolic fraction of allergic lung tissues (Fig. 2C, 2E) when normalized against β-actin, whereas ERβ protein levels were not significantly affected, by the Ova treatment, in all the subcellular fractions examined (Fig. 2E), except for the mitochondrial fraction (Fig 2A). The cytosolic and total homogenate’s (95 kDa) GR protein was absent from the mitochondrial fraction of both control and allergic mice indicating the absence of any cytosolic or nuclear contamination from the mitochondrial fractions (Fig. 2C). Moreover, the purity and enrichment of the subcellular fractions obtained were justified by the absence of the mitochondrial protein COX-I from the cytosolic fractions and the presence of a small portion of actin in the mitochondrial fractions that possibly corresponds to functional interaction of mitochondria with the cytoskeleton. Since the mitochondrial fraction was isolated from total lung tissue, and therefore originated from a mixture of cells, we decided to perform immunofluorescence studies to elucidate whether the reduction of steroid receptors in mitochondria correspond at least in part to the bronchial epithelial cell layer.

Lung sections from control and allergic mice were subjected to fixation methods that provide optimal preservation of mitochondrial structures, as was indicated by COX-1 staining, and immunostained against GR and ER.

Co-localization of GR and ERβ with the mitochondrial COX-I component of OXPHOS Complex IV was verified at the bronchial epithelial cell layer (as described in Supporting Information S3). Overlaid representative images, co-localised white pixels, and resulting scatter plots are presented in Figure 3A and 3B. Manders’ overlap coefficient [36], [37] of GR and COX-I (0.85 and 0.81 for control and allergic mice, respectively) revealed that GR co-localized with the mitochondrial COX-I from bronchial epithelia. Whereas, ERβ and COX-I Manders’ overlap coefficient was 0.85 and 0.83 for control and allergic mice, respectively. These high values of overlap coefficient indicate a strong pixels overlap between GR and COX-I as well as between ERβ and COX-I, revealing mitochondrial localization of GR (mtGR) and ER (mtER). The cytoplasmic and nuclear distribution of both GR and ERβ (Figures 3A and 3B, respectively) was also observed in triple stained sections, where the Hoechst fluorescence dye (blue) was used for nuclear staining.

Analysed immunofluorescence images revealed reduced mean intensity signal for both GR and ER in bronchial epithelial cell layer of allergic mice compared to controls (Figure 4A, 4B, 4C). Allergic airway inflammation also reduced mtGR by approximately 23% in allergic mice compared to controls (Figure 4A and 4D). Representative pictures showing co-localised GR with COX-I are presented as white pixels in Figure 4A. However, inflammatory cell infiltrates, which are characteristic in allergic mice, expressed increased GR at the inflammatory foci (Figure S2). In addition, allergic airway inflammation significantly reduced mtERβ by approximately 33% in allergic mice compared to controls (Figure 4B and 4D). Co-localized pixel showing mtERβ are demonstrated as white pixels (Figure 4B).

Similar analysis of GR and ERβ colocalization with the Hoechst dye revealed that the nuclear localization of GR was not significantly affected by allergic airway inflammation, whereas the nuclear localization of ERβ was also reduced by Ova treatment (Fig. 4E).

### Asthma Reduces mtGR and mtER in Human Bronchial Epithelial Cells

In order to validate our findings in human asthma, we studied the presence of mtGR and mtERβ in bronchial epithelial cell layer from autopsies sections of fatal asthma patients. Representative overlaid images of GR-COX-I and ERβ-COX-I and resulting co-localised white pixels are presented in Figure 5A and 5B. The co-localized white pixels were quantified in fatal asthmatic patients and results were expressed as percentage of control cases. mtGR and mtER were reduced in bronchial epithelial cell layer of patients fatal asthma by approximately 46% and 63%, respectively (Figure 5C). Because translocation of GR to mitochondria is demonstrated to correlate with susceptibility to CS-induced apoptosis in certain type of cells [38], [39], and apoptosis is increased in asthmatic bronchial epithelial cell [14], [40], we decided to look into the pro-apoptotic mitochondrial signalling in our mouse model of allergic airway inflammation.

### Allergic Airway Inflammation Increases Pro-apoptotic Mitochondrial Signalling at the Bronchial Epithelial Layer

Allergic airway inflammation induced significantly increased release of mitochondrial pro-apoptotic cytochrome c into the lung cell cytosol of allergic mice compared to controls (Figure 6A and 6B). The release of cytochrome c leads to activation of caspase-9, as demonstrated by the reduction in un-cleaved caspase-9 level in the cytosol of allergic mice compared to controls (Figure 6A and 6B). Subsequently, caspase-3 becomes activated, as illustrated in Figure 6C and 6D by the significantly increased production of cleaved caspase-3 in allergic mice compared to controls. An additional evidence for apoptosis activation is provided by the significant reduction in the level of the DNA repair enzyme PARP in allergic mice compared to controls (Figure 6C and 6D). Moreover, confocal images of cleaved caspase-3 in mice lung sections revealed predominant production of cleaved caspase-3 at the epithelial layer of allergic mice compared to controls (Figure 6E), as shown by either the red pixels in the overlay images or the white pixels in the grey scale image.

## Discussion

In the present study we used a mouse model of acute allergic airway disease to investigate the presence of GRα and ERβ in lung mitochondria, and the possible role of these putative mitochondrial receptors in allergic airway inflammation. We demonstrated the presence of GRα and ERβ in lung mitochondria and presented evidence showing that allergic airway inflammation reduces the presence of GR and ER in mitochondria of bronchial epithelial cells. Our findings was validated in a human disease as demonstrated by the significant reduction in the presence of GR and ER in the mitochondria of human bronchial epithelial cells in lung sections from fatal asthma patients, further supporting that allergic airway inflammation affects the presence of these receptors in mitochondria.

The quantity of mitochondria in the whole lung has been studied by various groups, with variable results [27], [29]. However, the absolute percentage of reduction may vary among mice models of allergic airway inflammation studied [41]. In this study, applying independent techniques, we showed that allergic airway inflammation significantly reduced two mitochondrial OXPHOS enzymes, the nuclear encoded SDH, and the mitochondrial encoded COX-I in lung tissues. Likewise, total citrate synthase activity from allergic mice was decreased in comparison to the healthy controls. Furthermore, mouse lung sections immunostained with anti-COX-I and visualized by confocal microscopy allowed us to ascertain that COX-I was particularly reduced at the bronchial epithelial layer. Our results are in agreement with findings showing that allergic airway inflammation causes reduction in mitochondrial Complex IV in bronchial epithelial cells and mitochondrial dysfunction [27]. Others have observed that in human asthma, at the chronic phase of the disease, mitochondria are increased in the bronchial smooth muscle layer [29]. However, these results refer to muscle cells in a chronic model of asthma and should be interpreted accordingly.

Taking into consideration that OXPHOS enzyme biosynthesis and mitochondrial biogenesis is activated by both nuclear and mitochondrial steroid and thyroid hormone receptors [13], [18], [21] we examined a) the presence of GR and ERβ in subcellular fractions, and particularly in mitochondrial fraction, from lung tissues and b) the possible effect of allergic airway inflammation on the mitochondrial localization of the receptors. We demonstrated *in vivo* for the first time the presence of GRα and ERβ in lung mitochondria of bronchial epithelial cells. In addition, we observed that allergic airway inflammation causes an overall reduction of these receptors in mitochondrial fraction isolated from total lung tissue. A GRα isoform, of an approximately 80 kDa molecular weight, which could correspond to the GRα-Β or GRα-C isoform, resulting from alternative translation initiation site in a single GRα mRNA [42], was found in a purified mitochondrial sub-fraction. This isoform, as it is previously suggested [25], [33], may also represent a functional proteolytic product of an inducible endoprotease activation, which leads to uncovering cryptic mitochondrial targeting signals and to mitochondrial translocation of the product [33], [38]. Despite the absence of the classical mitochondrial import sequences from the glucocorticoid receptor molecule, in silico analysis revealed the presence of such cryptic mitochondrial targeting signals, of alpha helical structures, in the C-terminal of glucocorticoid, estrogen, and androgen receptor [33]. Recently, Carazo et al [43] have proved that two alpha helix in the C-terminal part of the mitochondrial thyroid receptor (p43) represent actual import sequences, whose functionality depends also on the N-terminal region of p43. Taking into account the structural similarity of the nuclear receptor superfamily members, authors suggested that the proposed mechanism of the mitochondrial translocation of p43 could be extrapolated to other mitochondrial proteins related to the nuclear receptors superfamily, including GR and ERβ, pointing out the central role of both the N- and C- terminal domains of the molecules in the control of steroid/thyroid receptors mitochondrial targeting and regulation of mitochondrial function. Deregulation of the production of the functional mitochondrial targeted steroid receptor isoforms, under pathological conditions, such as in asthma, may result in the observed mtGR and mtERβ reduction in lung cells by allergic airway inflammation.

The GRβ isoform is totally absent in mice [44] and in mitochondria of various GRβ positive mammalian tissues [33]. Cytosolic GR (95 kD molecular weight protein) was increased by allergic airway inflammation possibly due to its presence in increased number of infiltrating inflammatory cells, whereas, it was absent from the mitochondrial fraction of both control and allegic mice, indicating the absence of any cytosolic contamination in mitochondrial fractions. Although to a lower extent, compared to mtGR reduction, mitochondrial ERβ was also decreased in allergic mice, whereas ERβ expression in total homogenate and cytosolic fraction was not significantly affected by allergic airway inflammation. The ERα isoform was not found in isolated mitochondria from allergic and control healthy mice, however, certain human bronchial epithelial cell lines express both mtERα and mtERβ [45].

The GRα and ERβ were studied both as relative mean fluorescence intensity and as percentage of co-localization rate, either with COX-I or Hoechst, in regions of interest (ROIs) placed at bronchial epithelial layer of mice lung sections. Results from these analyses provided evidence that allergic airway inflammation reduces both mtGRα and mtERβ in mice bronchial epithelial cell layer. Interestingly, nuclear localization of GR remained unaffected, whereas nuclear localization of ERβ was also decreased by allergic airway inflammation. In addition, we evaluated these findings in humans, where we demonstrated for the first time that bronchial epithelial layer from poorly-controlled and under-treated fatal asthma patients presents reduction in mtGR and mtERβ. As a limitation of our study, the inherent loss of intact epithelial layer, due to the disease and the post-mortem changes in controls and patients [32], constrained the performed analyses procedure. A fatal asthma attack can be considered an extremely severe acute exacerbation of the disease in a poorly controlled patient. The OVA model used in this study reflects an acute asthma model, caused by allergic sensitisation. However, it is very difficult to compare mice models of asthma with human asthma of different severities. Human asthma is a very heterogeneous disease, especially severe asthma, with differences in genetic backgrounds, environmental exposures, age of onset, presence of atopy, smoking, obesity, e.t.c [46]. In addition, mice have a different airway anatomy and partially different immune responses. Therefore, the extent to which findings in OVA model of mice can be transposed to the different spectra of human asthma is always subjected to caution.

Interestingly, reduction of glucocorticoid receptor mRNA and protein expression in the lungs of mice exposed to allergen has been reported [47]. This finding in combination with our observation of an overall decreased expression of steroid receptors in epithelial cells of allergic mice suggests that an overall reduction in GR and ER may also compromised asthma immune response. Nevertheless, the observed exclusively compartmentalized reduction of GR in mitochondria suggests a crucial role of mtGR in this process. In addition, the pronounced mtGR reduction in allergic mice compared to other mitochondrial proteins examined suggests an important role of mtGR in the signalling events triggered by allergic airway inflammation.

Since the mitochondrial GR and ERβ localization has been correlated with the activation of mitochondrial transcription and OXPHOS enzyme biosynthesis in various type of cells [24], [25], [48], and alteration in OXPHOS enzyme biosynthesis have been associated with changes in mitochondrial steroid receptors levels [24], [48], one possible functional consequence for a reduced expression of these mitochondrial receptors in bronchial epithelial cells of asthmatic lung could be the reduction in OXPHOS enzyme biosynthesis. Deficiency in OXPHOS, bellow the demand, has been shown to potentially cause mitochondrial impairment, increased ROS production, and induction of apotosis in several type of cells [11], [49], [50].

Additionally, mtGRα has been demonstrated to be involved in the regulation of apoptotic and inflammatory processes [38], [39], [51]. Other studies have also revealed anti-apoptotic [21], [52], [53] and anti-inflammatory activities of mtERβ [54]. Thus, the reduced expression of both mtGR and mtERβ could indicate a reduced protection against apoptosis and inflammation [13], [21], [53], [54]. In our model system we observed that allergic airway inflammation triggered mitochondria activated apoptosis as demonstrated by increased expression of cytochrome c in the cytosol followed by activation of the caspase cascade (caspase-3 and 9) and degradation of PARP. Moreover, increased immune-reactivity for cleaved caspase-3 in lung sections from allergic mice demonstrated that allergic airway inflammation induces apoptosis predominantly at the bronchial epithelial cells. However, the direct link between reduction of mitochondrial steroid receptors and induction of epithelial cells apoptosis in asthma remains to be proved. Apoptosis and immune response are multi-component processes requiring contributions from both genomic and cytoplasmic signalling events. The central role of mitochondria in these processes is expanding and although the proposed involvement of the mitochondrial targeted nuclear receptors is emerging (reviewed in [12], [13]), the exact mechanism of their actions remains to be further elucidated.

In summary, our study reports the presence of mtGR and mtERβ in lung tissue. More interestingly, we showed reduction of these mitochondrial steroid receptors by allergic airway inflammation in lung cells, and particularly in bronchial epithelial cells, in an acute allergic airway disease mouse model and in a human disease. Since these receptors are important regulators of mitochondrial and cellular processes, critical for the final outcome of the disease in asthma, their reduction in asthma may contribute, at least in part, to bronchial epithelial cells damages, rendering them putative pharmaceutical targets.

## Supporting Information

Figure S1 **Allergic airway inflammation in mice sensitized and challenged with Ova.** Airway inflammation was assessed in mice sensitized with Ova (Allergic) and with alum (Control) as described in Methods section. (A) Differential cell counts in the bronchoalveolar lavage (BAL) of Allergic and Control mice. (B) Lungs were prepared for histology, stained with H&E or PAS and scored by a blinded observer. (C) Lung homogenates and plasma from allergic and control mice were analysed for the expression of IL-4 using ELISA as described in Methods section. (D) Newtonian resistance (Rn). Results are presented as means ± SEM. n = 8–12 mice per group; *p<0.05 from control, **p<0.01 from control, ***p<0.001 from control. Scale bars, 100 µm.
(TIF)

Figure S2 **Increased GR at the inflammatory foci of allergic mice.** Confocal immunofluorescence images of control and allergic mice lung sections immunostained for COX-I (green), GR (red), and Hoeschst (nuclear fluorescence stain, blue) were obtained and analysed using Leica Las-AF image system. Representative images of COX-I staining, GR staining, overlay mtGR-COX-I- Hoeschst, and analysed co-localized mtGR-COX-I white pixels are depicted. White arrows indicate increased GR at the inflammatory foci of allergic mice compared to control mice. Scale bars, 50 µm.
(TIF)

Supporting Information S1 **Animals and experimental protocol.** Female BALB/c mice, 8–12 wk were sensitized with 0.01 mg ovalbumin (Ova) (Sigma-Aldrich) in 0.2 ml alum (Serva) intraperiponeally (i.p.) on days 0 and 12. Control mice received PBS/alum. Challenges were performed with aerosolized OVA (5%, for 20 min) days 18 to 21 [31]. Differential counts in Bronchoalveolar lavage (BAL) fluid were made on Wright-Geimsa-stained cytospins as previously described [31], [55]. Lung paraffin-embedded sections were stained with hematoxylin/eosin (H&E) or Periodic acid-schiff (PAS), as previously described [31], [55]. Levels of IL-4 (R&D Systems) in lung tissue homogenates were measured by ELISA as previously described [31], [55] according to the manufacturer’s instructions. Airway hyperresponsiveness (AHR) was measured as changes in Zrs using a modification of the low frequency forced-oscillation technique (LFOT) [55]. Anaesthetized mice were connected to a mechanical ventilator (Flexivent, SCIREQ, Montreal, Canada) and the constant phase model [56] was fit to the real and imaginary parts of the Zrs spectrum allowing the calculation of Newtonian airway resistance (Rn) as previously described [30], [55].
(DOC)

Supporting Information S2 **Subcellular fractionation for isolation of highly purified mitochondria.** Mice fresh lung were pool together and were homogenized in 3 volume of homogenization buffer (0.32 M sucrose, 5 mM Hepes, pH 7.4, 5 mM EDTA, 0.15 mM PMSF, 2 mM DTT, 2 µg/ml aprotinin, 5 µg/ml peptatin, and 1.25 µg/ml leupeptin) with 10 strokes of a glass teflon Potter-Elvejhem homogenizer, to give the total homogenates (TH) as previously described [33]. The homogenates were centrifuged for 5 min, at 1000 xg to give a pellet (P1) containing nuclei, unbroken cells, and cell debris and a low-speed supernatant (S1). S1 was centrifuged at 10,000 xg to provide the crude mitochondrial pellet (P2) and the post mitochondrial supernatant (S2). The P2 was washed once in homogenization buffer B (20 mM Tris pH 7.5, 0.07 M sucrose, 0.21 M mannitol, 1.5 mM MgCl_2_, 2.5 mM EDTA, 2.5 mM EGTA), re-suspended in the same buffer, layered onto a discontinuous sucrose gradient 1.0 M and 1.5 M sucrose and centrifuged at 64,000 xg for 30 min in a Sorval 5C ultra-centrifuge using a TH 641 rotor. Mitochondria were isolated from the interphase of 1.5 M and 1.0 M sucrose, diluted with three volumes of buffer C (20 mM Hepes pH 7.5, 1 mM EDTA, 1 mM EGTA) and subsequently centrifuged at 12,000 xg, for 20 min. The resulting pellet was washed with homogenization buffer B twice at 12,000 xg, for 20 min. The final pellet was kept at −80°C. S2 was centrifuged at 100,000 xg for 1 h in a Sorval 5C ultra-centrifuge using a T8100 rotor to give the soluble cytosol fraction.
(DOC)

Supporting Information S3 **Confocal laser scan microscopy analysis.** Fluorescence-stained sections were examined applying confocal laser scanning microscopy (Leica TCS SP5). Triple-stained images were obtained by sequential scanning for each channel to eliminate the “cross-talk” of chromophores and to ensure reliable quantification of co-localization. Quantitative analyses were performed employing the Leica LAS-AF image analysis, where a Region-of-interest (ROI) was manually selected using lasso tool. In a selected ROI, measurements of the relative mean intensity of the fluorescence signals were taken by the Leica LAS-AF image analysis programme. For colocalization stydies, in a selected ROI, images were overlaid revealing the co-localized pixels, threshold and background corrections were set based on Red-Green or Red-blue scatter gram. After setting background and threshold, % co-localization rate was calculated by Leica LAS-AF image analysis (% co-localization rate  =  co-localization area/area foreground; area foreground  =  area image - area background), and labelled as white pixels. Manders’ overlap coefficient [36], [37] was also calculated by the program.
(DOC)

## References

[1] Vignola AM, Chanez P, Campbell AM, Souques F, Lebel B (1998). Airway inflammation in mild intermittent and in persistent asthma.. Am J Respir Crit Care Med.

[2] Riedl MA, Nel AE (2008). Importance of oxidative stress in the pathogenesis and treatment of asthma.. Curr Opin Allergy Clin Immunol.

[3] Walsh GM, Sexton DW, Blaylock MG (2003). Corticosteroids, eosinophils and bronchial epithelial cells: new insights into the resolution of inflammation in asthma.. J Endocrinol.

[4] Hulsmann AR, Raatgeep HR, den Hollander JC, Stijnen T, Saxena PR (1994). Oxidative epithelial damage produces hyperresponsiveness of human peripheral airways.. Am J Respir Crit Care Med.

[5] Bucchieri F, Puddicombe SM, Lordan JL, Richter A, Buchanan D (2002). Asthmatic bronchial epithelium is more susceptible to oxidant-induced apoptosis.. Am J Respir Cell Mol Biol.

[6] Alzoghaibi MA, Bahammam AS (2007). Lipid peroxides in stable asthmatics receiving inhaled steroids and long-acting beta2 -agonists.. Respirology.

[7] Diaz-Sanchez D, Tsien A, Fleming J, Saxon A (1999). Effect of topical fluticasone propionate on the mucosal allergic response induced by ragweed allergen and diesel exhaust particle challenge.. Clin Immunol.

[8] Modig L, Toren K, Janson C, Jarvholm B, Forsberg B (2009). Vehicle exhaust outside the home and onset of asthma among adults.. Eur Respir J.

[9] Janssen-Heininger YM, Poynter ME, Aesif SW, Pantano C, Ather JL (2009). Nuclear factor kappaB, airway epithelium, and asthma: avenues for redox control.. Proc Am Thorac Soc.

[10] Evans RM (2005). The nuclear receptor superfamily: a rosetta stone for physiology.. *Mol Endocrinol*.

[11] Kroemer G, Reed JC (2000). Mitochondrial control of cell death.. *Nat Med*.

[12] Goldenthal MJ, Marin-Garcia J (2004). Mitochondrial signalling pathways: a receiver/integrator organelle.. Mol Cell Biochem.

[13] Psarra A-MG, Sekeris CE (2008). Nuclear receptors and other nuclear transcription factors in mitochondria: regulatory molecules in a new environment.. Biochim Biophys Acta.

[14] Dorscheid DR, Wojcik KR, Sun S, Marroquin B, White SR (2001). Apoptosis of airway epithelial cells induced by corticosteroids.. Am J Respir Crit Care Med.

[15] White SR, Dorscheid DR (2002). Corticosteroid-induced apoptosis of airway epithelium: a potential mechanism for chronic airway epithelial damage in asthma.. Chest.

[16] Adcock IM, Gilbey T, Gelder CM, Chung KF, Barnes PJ (1996). Glucocorticoid receptor localization in normal and asthmatic lung.. Am J Respir Crit Care Med.

[17] Korn SH, Wouters EF, Wesseling G, Arends JW, Thunnissen FB (1997). In vitro and in vivo modulation of alpha- and beta-glucocorticoid-receptor mRNA in human bronchial epithelium.. Am J Respir Crit Care Med.

[18] Montoya J, López-Pérez MJ, Ruiz-Pesini E (2006). Mitochondrial DNA transcription and diseases: past, present and future.. Biochim Biophys Acta.

[19] Enriquez JA, Fernandez-Silva P, Garrido-Perez N, Lopez-Perez MJ, Perez-Martos A (1999). Direct regulation of mitochondrial RNA synthesis by thyroid hormone.. Mol Cell Biol.

[20] Casas F, Rochard P, Rodier A, Cassar-Malek I, Marchal-Victorion S (1999). A variant form of the nuclear triiodothyronine receptor c-ErbAalpha1 plays a direct role in regulation of mitochondrial RNA synthesis.. Mol Cell Biol.

[21] Chen JQ, Cammarata PR, Baines CP, Yager JD (2009). Regulation of mitochondrial respiratory chain biogenesis by estrogens/estrogen receptors and physiological, pathological and pharmacological implications.. Biochim Biophys Acta.

[22] Sekeris CE (1990). The mitochondrial genome: a possible primary site of action of steroid hormones.. In Vivo.

[23] Demonacos C, Djordjevic-Markovic R, Tsawdaroglou N, Sekeris CE (1995). The mitochondrion as a primary site of action of glucocorticoids: the interaction of the glucocorticoid receptor with mitochondrial DNA sequences showing partial similarity to the nuclear glucocorticoid responsive elements.. J Steroid Biochem Mol Biol.

[24] Psarra A-MG, Sekeris CE (2011). Glucocorticoids induce mitochondrial gene transcription in HepG2 cells. Role of the mitochondrial glucocorticoid receptor.. Biochim Biophys Acta.

[25] Psarra A-MG, Sekeris CE (2009). Glucocorticoid receptors and other nuclear transcription factors in mitochondria and possible functions.. Biochim Biophys Acta.

[26] Chen JQ, Eshete M, Alworth WL, Yager JD (2004). Binding of MCF-7 cell mitochondrial proteins and recombinant human estrogen receptors alpha and beta to human mitochondrial DNA estrogen response elements.. J Cell Biochem.

[27] Mabalirajan U, Dinda AK, Kumar S, Roshan R, Gupta P (2008). Mitochondrial structural changes and dysfunction are associated with experimental allergic asthma.. J Immunol.

[28] Saffar AS, Alphonse MP, Shan L, HayGlass KT, Simons FER (2007). IgE modulates neutrophil survival in asthma: role of mitochondrial pathway.. J Immunol.

[29] Trian T, Benard G, Begueret H, Rossignol R, Girodet P-O (2007). Bronchial smooth muscle remodeling involves calcium-dependent enhanced mitochondrial biogenesis in asthma.. J Exp Med.

[30] Simoes DC, Xanthou G, Petrochilou K, Panoutsakopoulou V, Roussos C (2009). Osteopontin deficiency protects from airway remodeling and hyperresponsiveness in chronic asthma.. Am J Respir Crit Care Med.

[31] Xanthou G, Alissafi T, Semitekolou M, Simoes DC, Economidou E (2007). Osteopontin has a crucial role in allergic airway disease through regulation of dendritic cell subsets.. Nat Med.

[32] Green FH, Williams DJ, James A, McPhee LJ, Mitchell I (2010). Increased myoepithelial cells of bronchial submucosal glands in fatal asthma.. Thorax.

[33] Psarra A-MG, Solakidi S, Trougakos IP, Margaritis LH, Spyrou G (2005). Glucocorticoid receptor isoforms in human hepatocarcinoma HepG2 and SaOS-2 osteosarcoma cells: presence of glucocorticoid receptor alpha in mitochondria and of glucocorticoid receptor beta in nucleoli.. Int J Biochem Cell Biol.

[34] Solakidi S, Psarra A-MG, Nikolaropoulos S, Sekeris CE (2005). Estrogen receptors alpha and beta (ERalpha and ERbeta) and androgen receptor (AR) in human sperm: localization of ERbeta and AR in mitochondria of the midpiece.. Hum Reprod.

[35] Srere PA (1969). Citrate Synthase.. Methods Enzymol.

[36] Zinchuk V, Zinchuk O, Okada T (2007). Quantitative colocalization analysis of multicolor confocal immunofluorescence microscopy images: pushing pixels to explore biological phenomena.. Acta Histochem Cytochem.

[37] Manders EMM, Vernejoux J-M, Aten JA (1993). Measurement of co-localization of objects in dual-colour confocal images.. J Microsc.

[38] Sionov RV, Cohen O, Kfir S, Zilberman Y, Yefenof E (2006). Role of mitochondrial glucocorticoid receptor in glucocorticoid-induced apoptosis.. J Exp Med.

[39] Du J, Wang Y, Hunter R, Wei Y, Blumenthal R (2009). Dynamic regulation of mitochondrial function by glucocorticoids.. Proc Natl Acad Sci USA.

[40] Dorscheid DR, Low E, Conforti A, Shifrin S, Sperling AI (2003). Corticosteroid-induced apoptosis in mouse airway epithelium: Effect in normal airways and after allergen-induced airway inflammation.. J Allergy Clin Immunol.

[41] Kannan MS, Deshpande DA (2003). Allergic asthma in mice: what determines the phenotype?. Am J Physiol Lung Cell Mol Physiol.

[42] Lu NZ, Cidlowski JA (2004). The origin and functions of multiple human glucocorticoid receptor isoforms.. Ann N Y Acad Sci.

[43] Carazo A, Levin J, Casas F, Seyer P, Grandemange S (2012). Protein sequences involved in the mitochondrial import of the 3,5,3′-L-triiodothyronine receptor p43.. J Cell Physiol. In press.

[44] Otto C, Reichardt HM, Schutz G (1997). Absence of glucocorticoid receptor-beta in mice.. J Biol Chem.

[45] Ivanova MM, Mazhawidza W, Dougherty SM, Minna JD, Klinge CM (2009). Activity and intracellular location of estrogen receptors [alpha] and [beta] in human bronchial epithelial cells.. Mol Cell Endocrinol.

[46] de Carvalho-Pinto RM, Cukier A, Angelini L, Antonangelo L, Mauad T (2012). Clinical characteristics and possible phenotypes of an adult severe asthma population.. Respir Med.

[47] Bailey MT, Kinsey SG, Padgett DA, Sheridan JF, Leblebicioglu B (2009). Social stress enhances IL-1beta and TNF-alpha production by Porphyromonas gingivalis lipopolysaccharide-stimulated CD11b+ cells.. Physiol Behav.

[48] Chen JQ, Russo PA, Cooke C, Russo IH, Russo J (2007). ERbeta shifts from mitochondria to nucleus during estrogen-induced neoplastic transformation of human breast epithelial cells and is involved in estrogen-induced synthesis of mitochondrial respiratory chain proteins.. Biochim Biophys Acta.

[49] Turrens JF (2003). Mitochondrial formation of reactive oxygen species.. J Physiol.

[50] Ueda S, Masutani H, Nakamura H, Tanaka T, Ueno M (2002). Redox control of cell death.. Antioxid Redox Signal.

[51] Psarra A-MG, Hermann S, Panayotou G, Spyrou G (2009). Interaction of mitochondrial thioredoxin with glucocorticoid receptor and NF-kappaB modulates glucocorticoid receptor and NF-kappaB signalling in HEK-293 cells.. Biochem J.

[52] Hsieh YC, Yu HP, Suzuki T, Choudhry MA, Schwacha MG (2006). Upregulation of mitochondrial respiratory complex IV by estrogen receptor-beta is critical for inhibiting mitochondrial apoptotic signalling and restoring cardiac functions following trauma-haemorrhage.. J Mol Cell Cardiol.

[53] Yang S-H, Sarkar SN, Liu R, Perez EJ, Wang X (2009). Estrogen Receptor {beta} as a Mitochondrial Vulnerability Factor.. J Biol Chem.

[54] Catley MC, Birrell MA, Hardaker EL, de Alba J, Farrow S (2008). Estrogen receptor beta: expression profile and possible anti-inflammatory role in disease.. J Pharmacol Exp Ther.

[55] Simoes DC, Vassilakopoulos T, Toumpanakis D, Petrochilou K, Roussos C (2008). Angiopoietin-1 protects against airway inflammation and hyperreactivity in asthma.. Am J Respir Crit Care Med.

[56] Hantos Z, Daroczy B, Suki B, Nagy S, Fredberg JJ (1992). Input impedance and peripheral inhomogeneity of dog lungs.. J Appl Physiol.

