# Supplementary figures and images for: Glucocorticoid and Estrogen Receptors Are Reduced in Mitochondria of Lung Epithelial Cells in Asthma
Source: PLoS One. 2012 Jun 27;7(6):e39183. doi: 10.1371/journal.pone.0039183 (PMC3384641; doi:10.1371/journal.pone.0039183)

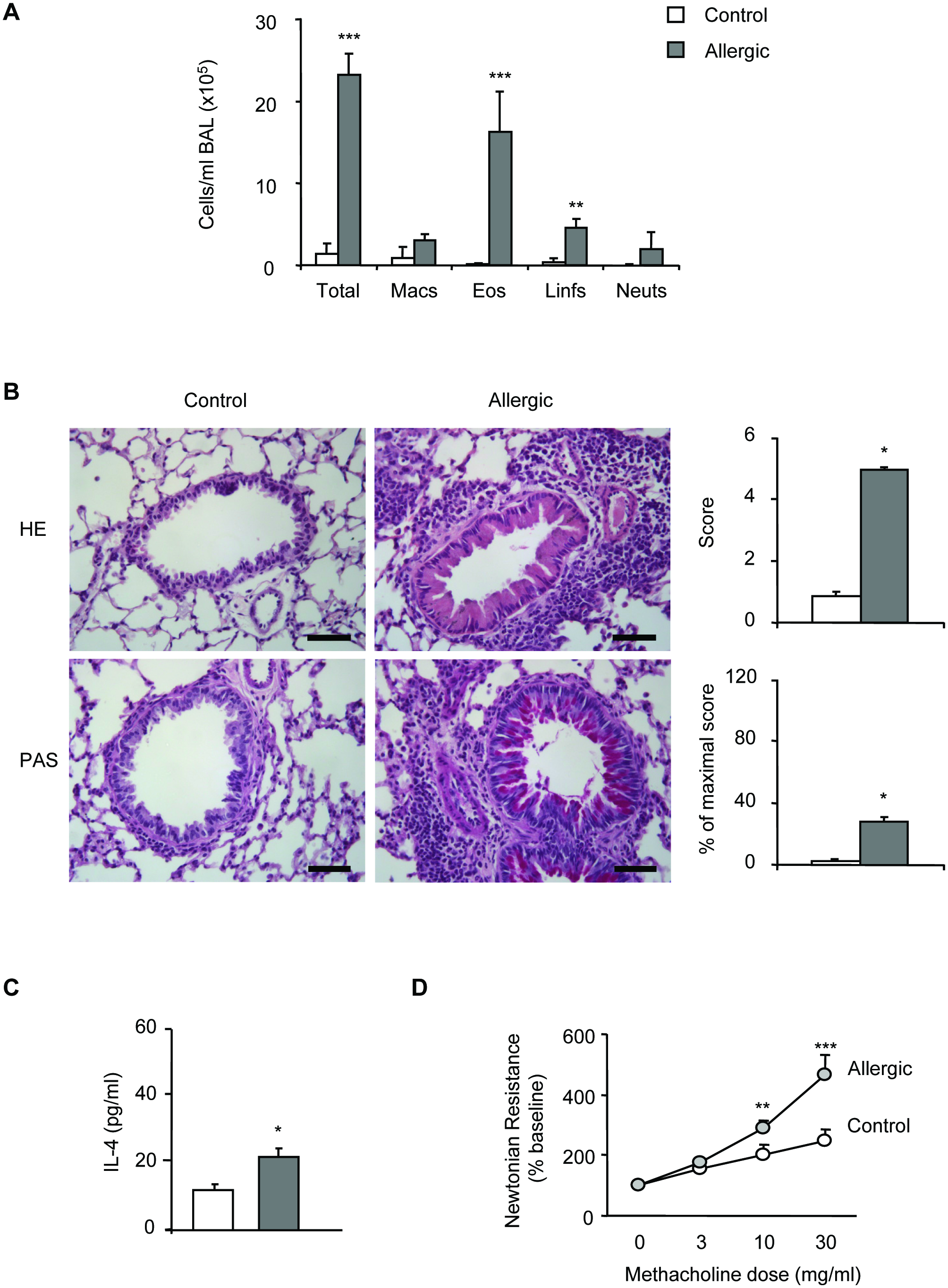

Supplement: Figure S1 — Allergic airway inflammation in mice sensitized and challenged with Ova. Airway inflammation was assessed in mice sensitized with Ova (Allergic) and with alum (Control) as described in Methods section. (A) Differential cell counts in the bronchoalveolar lavage (BAL) of Allergic and Control mice. (B) Lungs were prepared for histology, stained with H&E or PAS and scored by a blinded observer. (C) Lung homogenates and plasma from allergic and control mice were analysed for the expression of IL-4 using ELISA as described in Methods section. (D) Newtonian resistance (Rn). Results are presented as means ± SEM. n = 8–12 mice per group; *p<0.05 from control, **p<0.01 from control, ***p<0.001 from control. Scale bars, 100 µm. (TIF) [file pone.0039183.s001.tif]

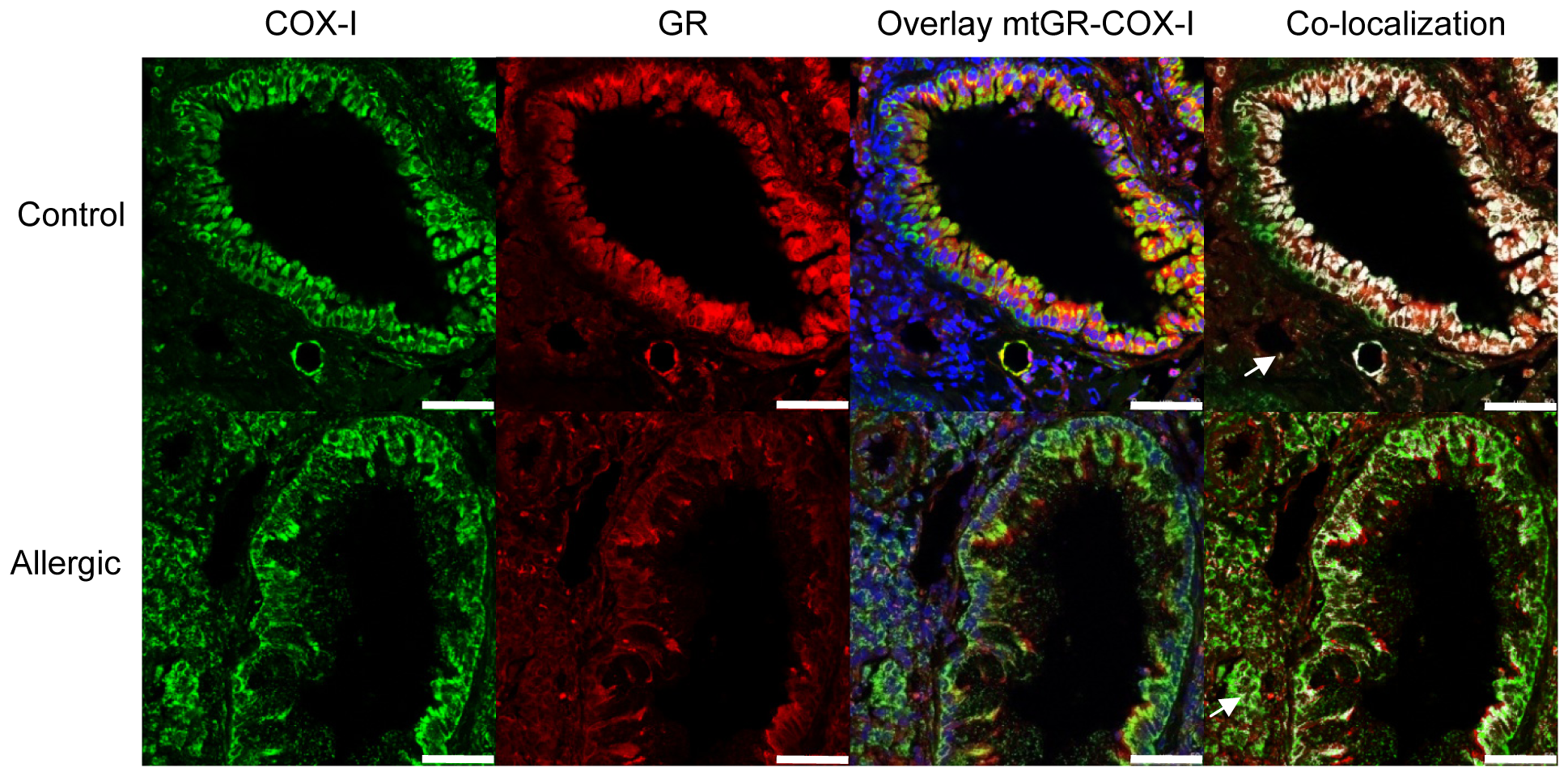

Supplement: Figure S2 — Increased GR at the inflammatory foci of allergic mice. Confocal immunofluorescence images of control and allergic mice lung sections immunostained for COX-I (green), GR (red), and Hoeschst (nuclear fluorescence stain, blue) were obtained and analysed using Leica Las-AF image system. Representative images of COX-I staining, GR staining, overlay mtGR-COX-I- Hoeschst, and analysed co-localized mtGR-COX-I white pixels are depicted. White arrows indicate increased GR at the inflammatory foci of allergic mice compared to control mice. Scale bars, 50 µm. (TIF) [file pone.0039183.s002.tif]
