# Supplementary material for: Glucocorticoid and Estrogen Receptors Are Reduced in Mitochondria of Lung Epithelial Cells in Asthma
Source: PLoS One. 2012 Jun 27;7(6):e39183. doi: 10.1371/journal.pone.0039183 (PMC3384641; doi:10.1371/journal.pone.0039183)
Supplement: Supporting Information S1 — Animals and experimental protocol. Female BALB/c mice, 8–12 wk were sensitized with 0.01 mg ovalbumin (Ova) (Sigma-Aldrich) in 0.2 ml alum (Serva) intraperiponeally (i.p.) on days 0 and 12. Control mice received PBS/alum. Challenges were performed with aerosolized OVA (5%, for 20 min) days 18 to 21 [31]. Differential counts in Bronchoalveolar lavage (BAL) fluid were made on Wright-Geimsa-stained cytospins as previously described [31], [55]. Lung paraffin-embedded sections were stained with hematoxylin/eosin (H&E) or Periodic acid-schiff (PAS), as previously described [31], [55]. Levels of IL-4 (R&D Systems) in lung tissue homogenates were measured by ELISA as previously described [31], [55] according to the manufacturer’s instructions. Airway hyperresponsiveness (AHR) was measured as changes in Zrs using a modification of the low frequency forced-oscillation technique (LFOT) [55]. Anaesthetized mice were connected to a mechanical ventilator (Flexivent, SCIREQ, Montreal, Canada) and the constant phase model [56] was fit to the real and imaginary parts of the Zrs spectrum allowing the calculation of Newtonian airway resistance (Rn) as previously described [30], [55]. (DOC) [file pone.0039183.s003.doc]

**Supporting Information S1. Animals and experimental protocol.** Female BALB/c mice, 8-12 wk were sensitized with 0.01 mg ovalbumin (Ova) (Sigma-Aldrich) in 0.2 ml alum (Serva) intraperiponeally (i.p.) on days 0 and 12. Control mice received PBS/alum. Challenges were performed with aerosolized OVA (5%, for 20 min) days 18 to 21 [31]. Differential counts in Bronchoalveolar lavage (BAL) fluid were made on Wright-Geimsa-stained cytospins as previously described [31,55]. Lung paraffin-embedded sections were stained with hematoxylin/eosin (H&E) or Periodic acid-schiff (PAS), as previously described [31,55]. Levels of IL-4 (R&D Systems) in lung tissue homogenates were measured by ELISA as previously described [31,55] according to the manufacturer’s instructions. Airway hyperresponsiveness (AHR) was measured as changes in Zrs using a modification of the low frequency forced-oscillation technique (LFOT) [55]. Anaesthetized mice were connected to a mechanical ventilator (Flexivent, SCIREQ, Montreal, Canada) and the constant phase model [56] was fit to the real and imaginary parts of the Zrs spectrum allowing the calculation of Newtonian airway resistance (Rn) as previously described [30,55].
