# Supplementary material for: Glucocorticoid and Estrogen Receptors Are Reduced in Mitochondria of Lung Epithelial Cells in Asthma
Source: PLoS One. 2012 Jun 27;7(6):e39183. doi: 10.1371/journal.pone.0039183 (PMC3384641; doi:10.1371/journal.pone.0039183)
Supplement: Supporting Information S2 — Subcellular fractionation for isolation of highly purified mitochondria. Mice fresh lung were pool together and were homogenized in 3 volume of homogenization buffer (0.32 M sucrose, 5 mM Hepes, pH 7.4, 5 mM EDTA, 0.15 mM PMSF, 2 mM DTT, 2 µg/ml aprotinin, 5 µg/ml peptatin, and 1.25 µg/ml leupeptin) with 10 strokes of a glass teflon Potter-Elvejhem homogenizer, to give the total homogenates (TH) as previously described [33]. The homogenates were centrifuged for 5 min, at 1000 xg to give a pellet (P1) containing nuclei, unbroken cells, and cell debris and a low-speed supernatant (S1). S1 was centrifuged at 10,000 xg to provide the crude mitochondrial pellet (P2) and the post mitochondrial supernatant (S2). The P2 was washed once in homogenization buffer B (20 mM Tris pH 7.5, 0.07 M sucrose, 0.21 M mannitol, 1.5 mM MgCl2, 2.5 mM EDTA, 2.5 mM EGTA), re-suspended in the same buffer, layered onto a discontinuous sucrose gradient 1.0 M and 1.5 M sucrose and centrifuged at 64,000 xg for 30 min in a Sorval 5C ultra-centrifuge using a TH 641 rotor. Mitochondria were isolated from the interphase of 1.5 M and 1.0 M sucrose, diluted with three volumes of buffer C (20 mM Hepes pH 7.5, 1 mM EDTA, 1 mM EGTA) and subsequently centrifuged at 12,000 xg, for 20 min. The resulting pellet was washed with homogenization buffer B twice at 12,000 xg, for 20 min. The final pellet was kept at −80°C. S2 was centrifuged at 100,000 xg for 1 h in a Sorval 5C ultra-centrifuge using a T8100 rotor to give the soluble cytosol fraction. (DOC) [file pone.0039183.s004.doc]

**Supporting Information S2. Subcellular fractionation for isolation of highly purified mitochondria.** Mice fresh lung were pool together and were homogenized in 3 volume of homogenization buffer (0.32 M sucrose, 5 mM Hepes, pH 7.4, 5 mM EDTA, 0.15 mM PMSF, 2 mM DTT, 2 µg/ml aprotinin, 5 µg/ml peptatin, and 1.25 µg/ml leupeptin) with 10 strokes of a glass teflon Potter-Elvejhem homogenizer, to give the total homogenates (TH) as previously described [33]. The homogenates were centrifuged for 5 min, at 1000xg to give a pellet (P1) containing nuclei, unbroken cells, and cell debris and a low-speed supernatant (S1). S1 was centrifuged at 10,000xg to provide the crude mitochondrial pellet (P2) and the post mitochondrial supernatant (S2). The P2 was washed once in homogenization buffer B (20 mM Tris pH 7.5, 0.07 M sucrose, 0.21 M mannitol, 1.5 mM MgCl_2_, 2.5 mM EDTA, 2.5 mM EGTA), re-suspended in the same buffer, layered onto a discontinuous sucrose gradient 1.0 M and 1.5 M sucrose and centrifuged at 64,000xg for 30 min in a Sorval 5C ultra-centrifuge using a TH 641 rotor. Mitochondria were isolated from the interphase of 1.5 M and 1.0 M sucrose, diluted with three volumes of buffer C (20 mM Hepes pH 7.5, 1 mM EDTA, 1 mM EGTA) and subsequently centrifuged at 12,000xg, for 20 min. The resulting pellet was washed with homogenization buffer B twice at 12,000xg, for 20 min. The final pellet was kept at -80°C. S2 was centrifuged at 100,000xg for 1h in a Sorval 5C ultra-centrifuge using a T8100 rotor to give the soluble cytosol fraction.
