# Supplementary material for: Glucocorticoid and Estrogen Receptors Are Reduced in Mitochondria of Lung Epithelial Cells in Asthma
Source: PLoS One. 2012 Jun 27;7(6):e39183. doi: 10.1371/journal.pone.0039183 (PMC3384641; doi:10.1371/journal.pone.0039183)
Supplement: Supporting Information S3 — Confocal laser scan microscopy analysis. Fluorescence-stained sections were examined applying confocal laser scanning microscopy (Leica TCS SP5). Triple-stained images were obtained by sequential scanning for each channel to eliminate the “cross-talk” of chromophores and to ensure reliable quantification of co-localization. Quantitative analyses were performed employing the Leica LAS-AF image analysis, where a Region-of-interest (ROI) was manually selected using lasso tool. In a selected ROI, measurements of the relative mean intensity of the fluorescence signals were taken by the Leica LAS-AF image analysis programme. For colocalization stydies, in a selected ROI, images were overlaid revealing the co-localized pixels, threshold and background corrections were set based on Red-Green or Red-blue scatter gram. After setting background and threshold, % co-localization rate was calculated by Leica LAS-AF image analysis (% co-localization rate = co-localization area/area foreground; area foreground = area image - area background), and labelled as white pixels. Manders’ overlap coefficient [36], [37] was also calculated by the program. (DOC) [file pone.0039183.s005.doc]

**Supporting Information S3:**

**Confocal laser scan microscopy analysis**

Fluorescence-stained sections were then examined applying confocal laser scanning microscopy (Leica TCS SP5). Triple-stained images were obtained by sequential scanning for each channel to eliminate the “cross-talk” of chromophores and to ensure reliable quantification of co-localization. Quantitative analyses were performed employing the Leica LAS-AF image analysis, where a Region-of-interest (ROI) was manually selected using lasso tool. In a selected ROI, measurements of the relative mean intensity of the fluorescence signals were taken by the Leica LAS-AF image analysis programme. For colocalization stydies, in a selected ROI, images were overlaid revealing the co-localized pixels, threshold and background corrections were set based on Red-Green or Red-blue scatter gram. After setting background and threshold, % co-localization rate was calculated by Leica LAS-AF image analysis (% co-localization rate = co-localization area/area foreground; area foreground = area image - area background), and labelled as white pixels. Manders’ overlap coefficient [36,37] was also calculated by the program
